# Habitat‐linked genetic structure for white‐crowned sparrow (*Zonotrichia leucophrys*): Local factors shape population genetic structure

**DOI:** 10.1002/ece3.7887

**Published:** 2021-08-10

**Authors:** Catherine A. Welke, Brendan Graham, Ross R. Conover, James W. Rivers, Theresa M. Burg

**Affiliations:** ^1^ Department of Biological Sciences University of Lethbridge Lethbridge AB Canada; ^2^ Department of Biology The King's University Edmonton AB Canada; ^3^ Department of Natural Sciences Paul Smith's College Paul Smiths New York USA; ^4^ Department of Forest Engineering, Resources, and Management Oregon State University Corvallis Oregon USA

**Keywords:** gene flow, habitat mediated dispersal, landscape genetics, microsatellites, population genetics, rangewide barriers, white‐crowned sparrow

## Abstract

Ecological, environmental, and geographic factors all influence genetic structure. Species with broad distributions are ideal systems because they cover a range of ecological and environmental conditions allowing us to test which components predict genetic structure. This study presents a novel, broad geographic approach using molecular markers, morphology, and habitat modeling to investigate rangewide and local barriers causing contemporary genetic differentiation within the geographical range of three white‐crowned sparrow (*Zonotrichia leucophrys*) subspecies: *Z. l. gambelii, Z. l. oriantha,* and *Z. l. pugetensis*. Three types of genetic markers showed geographic distance between sampling sites, elevation, and ecosystem type are key factors contributing to population genetic structure. Microsatellite markers revealed white‐crowned sparrows do not group by subspecies, but instead indicated four groupings at a rangewide scale and two groupings based on coniferous and deciduous ecosystems at a local scale. Our analyses of morphological variation also revealed habitat differences; sparrows from deciduous ecosystems are larger than individuals from coniferous ecosystems based on principal component analyses. Habitat modeling showed isolation by distance was prevalent in describing genetic structure, but isolation by resistance also had a small but significant influence. Not only do these findings have implications concerning the accuracy of subspecies delineations, they also highlight the critical role of local factors such as habitat in shaping contemporary population genetic structure of species with high dispersal ability.

## Graphical abstract

In this study, we investigate the effect of rangewide and local barriers on contemporary genetic differentiation for western populations of white‐crowned sparrows (*Zonotrichia leucophrys*). Microsatellite markers revealed that habitat influences genetic structure, as individuals found in coniferous ecosystems were genetically distinct from individuals found in deciduous habitats. Our results highlight the critical role of local ecosystem factors in shaping contemporary population genetic structure of species with high dispersal ability.

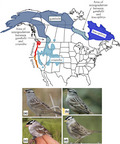

## 1 INTRODUCTION

Dispersal of individuals from one habitat patch to another is a simple concept, yet dispersal can have a profound effect on gene flow, genetic diversity, adaptation, and population dynamics (Bowler & Benton, 2005; Olah et al., 2017; Ronce, 2007). This is especially true for species that exhibit habitat specialization or low mobility where environmental and landscape features, including climate, habitat, and elevation, can act as barriers to dispersal (Geffen et al., 2004; Kershenbaum et al., 2014). For these reasons, the influence of landscape on population connectivity and genetic structure is complex and depends on individual behavior and the landscape composition (Coulon et al., 2004; Holderegger & Wagner, 2008; Lee‐Yaw et al., 2009; Rissler, 2016).

The relationship between gene flow and dispersal potential is not always apparent. Many species capable of flight are thought to have high population connectivity simply because they can cross or circumvent physical barriers such as anthropogenic development, large bodies of water, or mountain ranges, but this is not always true (Holderegger & Wagner, 2008). With the advent of landscape genetics, more studies show genetic differentiation can occur within contiguous populations due to microclimates and behavioral differences unrelated to physical barriers (Dubay & Witt, 2014; Engler et al., 2014; Porlier et al., 2012). For example, mitochondrial DNA differentiation exists between rufous‐collared sparrow (*Zonotrichia capensis*) populations locally adapted to low versus high elevation microclimates on the same mountain slope (Cheviron & Brumfield, 2009). Similarly, in the absence of bluegill sunfish (*Lepomis macrochirus*), some pumpkinseed sunfish (*Lepomis gibbosus*) changed from preying on snails to feeding on *Daphnia,* which resulted in morphological differences between the ecotypes (Robinson et al., 1993). Therefore, even in the presence of high dispersal potential and in the absence of conspicuous physical barriers, population genetic structure can occur at small spatial and short temporal scales (Porlier et al., 2012).

Physical barriers such as mountains are an effective barrier to dispersal for many species (Fjeldsâ et al., 2011; Hooge, 2003; Robertson et al., 2009). However, if a species with breeding sites on either side of a mountain range share a wintering ground and have low philopatry, this could lead to the incorrect conclusion that the mountains do not act as a barrier. In addition to physical barriers, environmental and habitat variables also affect genetic variation (Sexton et al., 2014). For example climate, habitat and diet have been found to influence genetic variation for a wide variety of aquatic and terrestrial species (Pilot et al., 2006; Selkoe et al., 2010; MacDonald et al., 2020). When gene flow is restricted between habitats or ecosystems, it can lead to the formation of genetically distinct ecotypes (Kess et al., 2018; Parchman et al., 2016).

**FIGURE 1:**
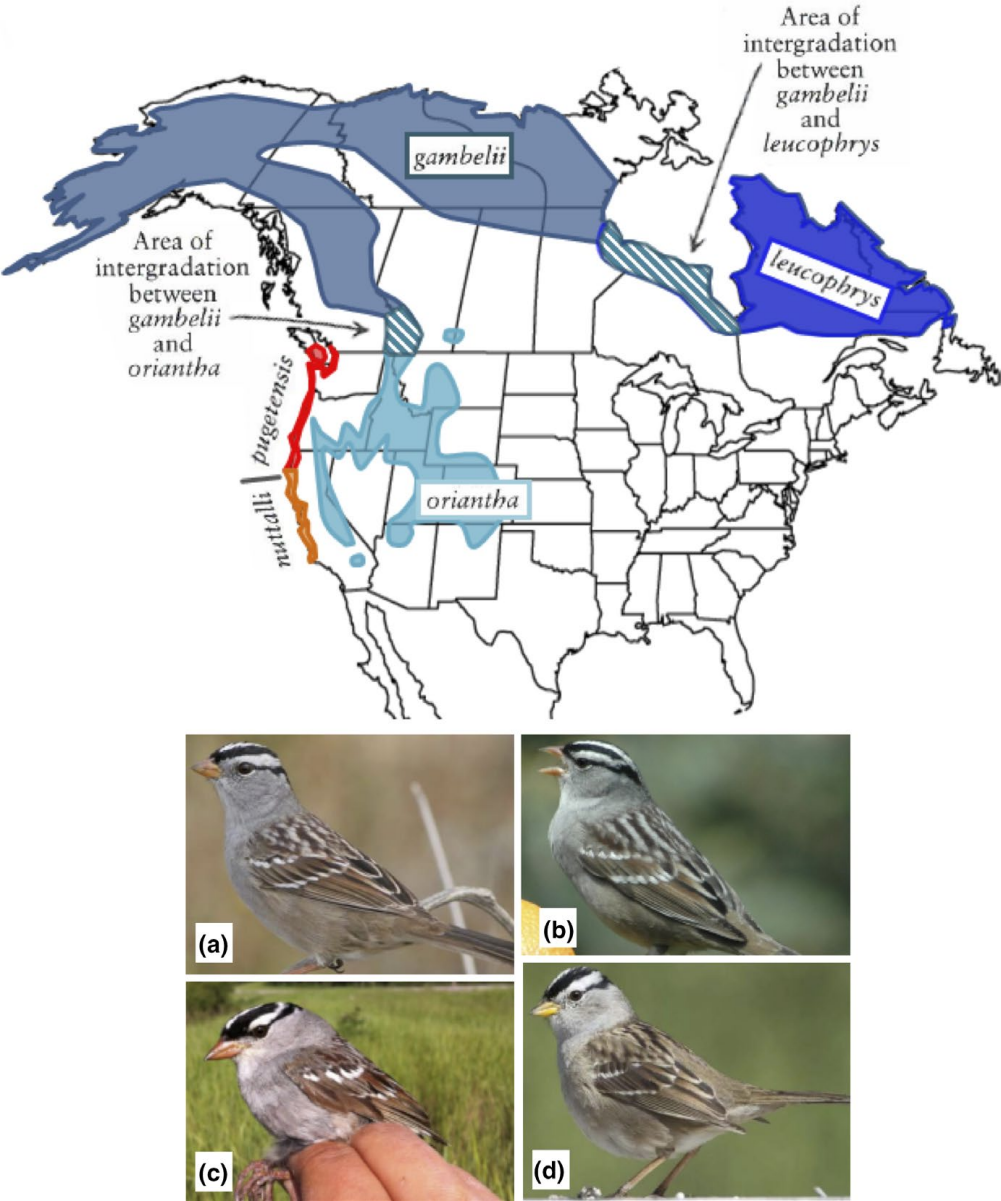
Breeding season range map of white‐crowned sparrow subspecies, *Z. l. gambelii, Z. l. leucophrys,* and *Z. l. oriantha* in shades of blue, *Z. l. pugetensis* in red, and *Z. l. nuttalli* in orange. Areas with cross‐hatching indicate areas of overlap between subspecies. Adapted from Dunn et al. (1995). Example photographs of subspecies show the following: (a) *Z. l. gambelii* has pale lores (small feathers between bill and eye) and orange bill. (b) *Z. l. leucophrys* has a thin line of black in the lores near the eye and a light pink bill. (c) *Z. l. oriantha* is very similar in appearance to *Z. l. leucophrys,* but has flared crown stripes and heavy black lores, a dusky pink bill with darker red on culmen (upper bill) and on average has a smaller body mass, longer wings, and shorter tarsi. (d) *Z. l. pugetensis* has pale lores and a yellow bill. *Z. l. nuttalli* is virtually indistinguishable in the field from *Z. l. pugetensis* (Dunn et al., 1995; Morton, 2002). Photo credits to C. Welke © and Don Robinson © (a, b, and d; with permission)

The white‐crowned sparrow (*Zonotrichia leucophrys*) is an ideal species for testing predictions about the effects of rangewide and local barriers on genetic structure. It is a widespread North American passerine whose range spans multiple barrier types and is composed of five subspecies with different song dialects, habitat preferences, phenotype, and migration behaviors (Figure 1). Previous studies suggest levels of gene flow differ among subspecies (Austen & Handford, 1991; Chilton et al., 1990; Lein & Corbin, 1990; Soha et al., 2004) and hybridization has been reported between two *Z. l. gambelii* and *Z. l. oriantha* (Lein & Corbin, 1990). To date, no studies have extensively examined the factors contributing to genetic variation within this species. Do genetic patterns arise as a result of environment or as a result of isolation by distance (Sexton et al., 2014)? In this study, we use genetic and environmental data for white‐crowned sparrows to examine how landscape composition and habitat mediate gene flow.

We examined population genetic structure for three subspecies *Z. l. pugetensis, Z. l. oriantha,* and *Z. l. gambelii* of the white‐crowned sparrow. We sampled the northern Rocky Mountains, a broad north–south belt of mountains interspersed with rift valleys and glacier‐carved basins (Cruden & Hu, 1999; Holland, 1976). *Z. l. oriantha* populations transition *to Z. l. gambelii* along a north–south habitat gradient, and coniferous alpine forests are replaced by riparian deciduous forests. We used genetic, landscape, and environmental data to complete three objectives examining how habitat and ecology mediate gene flow including (a) how geographic distance and the rangewide barriers (such as the Rocky Mountains) influence genetic structure; (b) whether habitat and microclimate variation act as local barriers to gene flow; and (c) if suitable habitat corridors mediate gene flow for white‐crowned sparrows.

## 2 MATERIALS AND METHODS

### 2.1 Study species and sample collection

**FIGURE 2:**
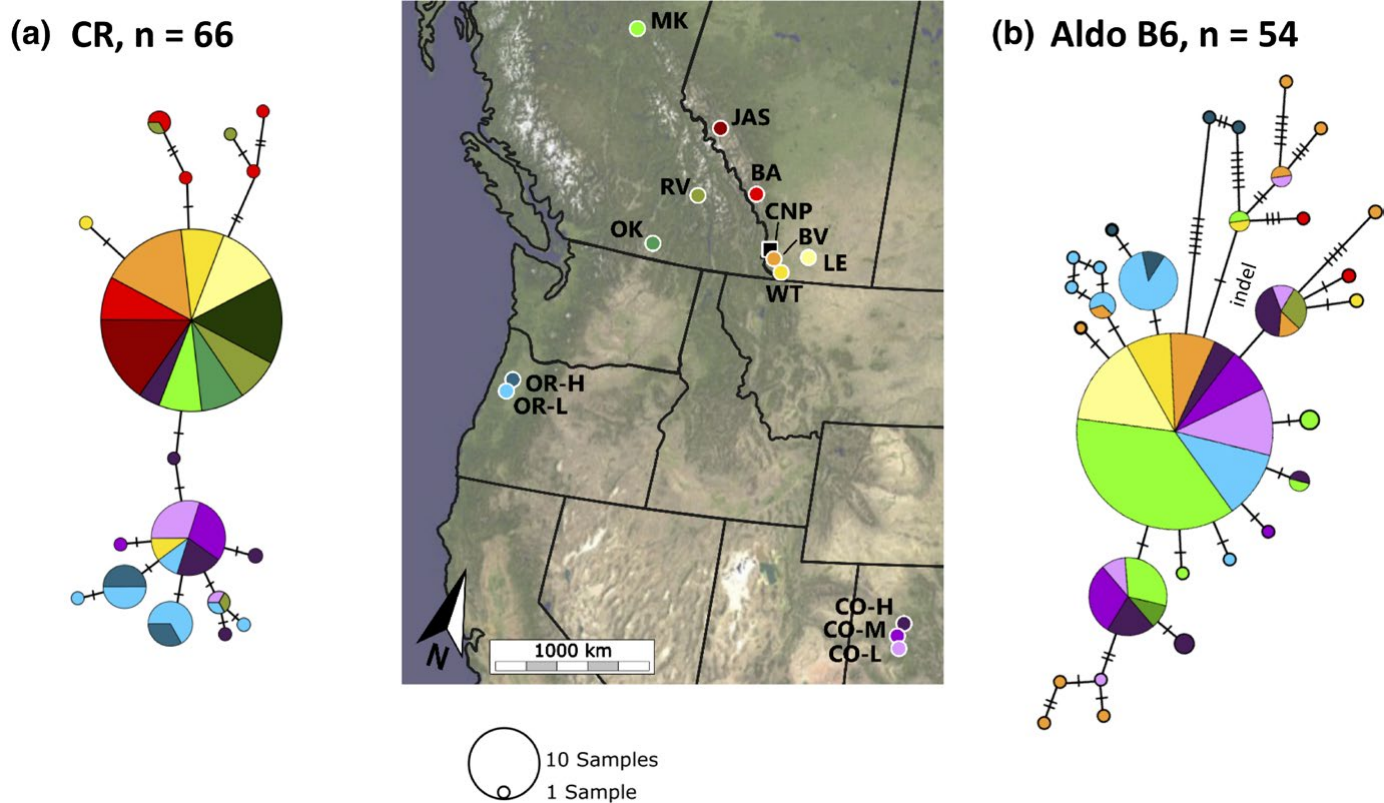
Map of the 15 sampling sites in this study and minimum spanning networks for CR using sequences from all sites except CNP and 66 birds (a) and AldoB6 using sequences from 12 sites and 54 birds (85 sequences after phasing male haplotypes) (b). Each circle represents a haplotype, with the number of dashes across connecting lines showing number of base pair differences. Colors correspond to the haplotype's population of origin. A 9 bp insertion linked to a 19 bp deletion in some AldoB6 haplotypes is indicated by “indel.” The single sample from OK is displayed in the network but omitted from *F*_ST_ comparisons. Site abbreviations available in Table 1

Five subspecies of white‐crowned sparrows form two groups: (a) the *Z. l. pugetensis* and *Z. l. nuttalli* populations breed on the Pacific Coast and (b) the *Z. l. leucophrys –oriantha–gambelii* populations breed to the east throughout North America (Figure 1). We sampled 328 adult white‐crowned sparrows from 15 populations (Table 1; Figure 2) representing three of the recognized subspecies (*Z. l. oriantha, Z. l. gambelii,* and *Z. l. pugetensis*). Birds were captured with mist‐nets using song playback over two breeding seasons (2015–2016; Figure 2), and a feather or small blood sample was taken from the brachial vein of each bird and stored in 99% ethanol except for in Oregon, where samples were collected in 2013–2014 and stored in Longmire buffer. We banded individuals with a numbered USGS metal band to prevent resampling and collected six morphometric measurements: bill length, bill depth, bill width, tarsus length, uncompressed wing chord, and total mass, which we used to compare morphological differences. Of the 328 individuals sampled, we obtained 28 tissue samples from the Royal Alberta and Royal British Columbia museums to supplement field sampling.

**TABLE 1:** Number of samples sequenced (*n*) at each sampling site and for each subspecies and location (ID) for control region (CR) and Aldolase B (AldoB6) sequences and microsatellite genotypes

Population	ID	CR sequences			AldoB6 sequences			Microsatellites					
		*n*	Hn	Hd	n*	Hn	Hd	*n*	Na	PA	AR	Ho	He
Jasper National Park	JAS	4	1	0.00	–	–	–	6	3.89	1	3.10	0.75	0.64
Banff National Park	BA	7	5	0.90	2	2	1.00	7	4.56	0	3.32	0.71	0.64
Beaver Mines	BV	4	1	0.00	12	11	0.98	13	5.67	2	3.93	0.58	0.69
Crowsnest Pass	CNP	–	–	–	–	–	–	10	5.33	2	3.71	0.60	0.62
Waterton Lakes National Park	WT	4	3	0.83	4	3	0.83	19	6.44	3	4.13	0.60	0.70
Lethbridge	LE	3	1	0.00	4	1	0.00	21	7.22	2	4.05	0.68	0.67
Fort St. James	FTSJ	4	1	0.00	–	–	–	11	3.44	0	2.70	0.47	0.52
Revelstoke	RV	5	4	0.90	2	1	0.00	4	3.33	0	3.02	0.59	0.54
Okanagan	OK	2	1	0.00	1	1	0.00	4	2.33	0	2.14	0.43	0.39
Mackenzie	MK	2	1	0.00	18	6	0.68	29	7.44	2	4.21	0.64	0.68
Colorado—Low elevation	CO‐L	4	2	0.50	7	5	0.83	23	7.44	3	4.32	0.74	0.73
Colorado—Mid elevation	CO‐M	4	2	0.50	6	3	0.73	32	7.22	2	4.56	0.67	0.70
Colorado—High elevation	CO‐H	7	5	0.90	9	5	0.86	28	7.33	0	4.53	0.75	0.73
Oregon—Low elevation	OR‐L	11	6	0.84	16	7	0.81	61	8.44	2	4.01	0.65	0.71
Oregon—High elevation	OR‐H	5	2	0.60	4	4	1.00	60	8.44	4	4.01	0.61	0.71
Population total		66	17	0.80	85	30	0.93	328	5.90	23	3.72	0.63	0.64
*Z. l. gambelii*	GWCS	15	5	0.56	15	6	0.84	67	10.11	5	7.72	0.64	0.71
*Z. l. oriantha*	MWCS	24	9	0.76	47	19	0.91	115	11.44	13	7.76	0.70	0.76
*Z. l. pugetensis*	PSWS	16	8	0.83	20	10	0.90	121	9.78	12	7.80	0.63	0.71
Subspecies total		55	17	0.83	82	30	0.92	303	10.44	30	7.76	0.66	0.73

Note

The number of haplotypes (Hn) and haplotype diversity (Hd) were calculated in DnaSP v5. Microsatellite samples were screened at nine loci with average number of different alleles (Na), number of private alleles (PA), allelic richness (AR), observed heterozygosity (Ho), and expected heterozygosity (He) reported. For AldoB6 number of sequences, n* refers to the number of chromosomes screened.

**FIGURE 3:**
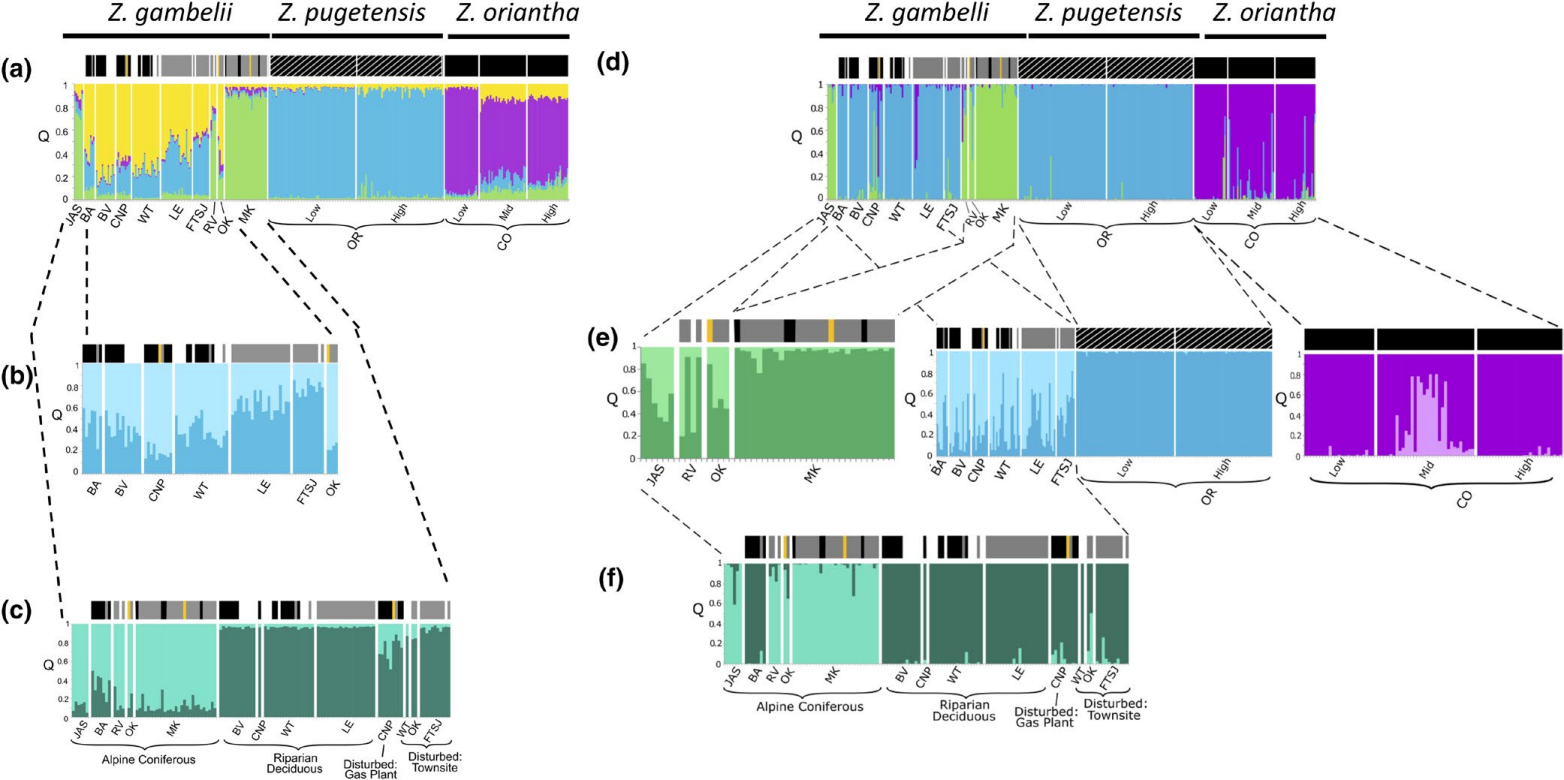
STRUCTURE plot of all populations with optimal number of genetic clusters of *K* = 4 as determined by highest log‐probability and Δ*K* for 328 samples (a). (b) Hierarchical analysis of the first group (yellow) had an optimal number of *K* = 2. (c) Substructure according to ecosite type for northern samples also had *K* = 2. Bayesian analysis using TESS v2.3 yielded an optimal *K* = 3 in the overall clustering (d), *K* = 2 for each of cluster 1 (light green), cluster 2 (light blue), Colorado elevation groups (purple) (e), and northern ecosite groups (f). Above each plot is a bar showing subspecies of each individual as *Z. l. gambelii* (gray), *Z. l. oriantha* (black), *Z. l. pugetensis* (striped), or hybrid *gambelii‐oriantha* (yellow). Unknown subspecies are uncolored

Of the 328 samples, 204 were collected at two sites to examine elevational differences (Table 1). A total of 83 blood samples were obtained from a 415m elevational transect north of the Rocky Mountain Biological Laboratory in Gunnison County, Colorado (38.96°N, −107.01°W) (CO). In Oregon, 121 blood samples were obtained from unrelated *Z. l. pugetensis* nestlings from four elevations in the Oregon Coast Range mountains (OR; Rivers et al., 2019). The birds at the lowest (25–100 m) and highest (400–500 m) elevations in four different OR sites were consolidated into two populations (OR‐L and OR‐H, respectively), and the CO birds grouped into low (~2,900 m), mid (~3,100 m), and high (3,330 m) elevations (CO‐L, CO‐M, and CO‐H, respectively) (Figure 2). We characterized the ecosystem types where samples were collected as alpine coniferous, riparian deciduous, disturbed‐gas plant, and disturbed‐town ecotypes (Figure 3). Ecosystems were characterized based on the dominant tree species and the degree of anthropogenic disturbance at each sampling site.

### 2.2 DNA extraction, amplification, and sequencing

Genomic DNA was extracted from blood–ethanol mix or Longmire buffer (10 µl), tissue (~1 µg), or feather samples (basal portion of feather shaft) using a modified Chelex procedure (Burg & Croxall, 2001; Walsh et al., 1991). We genotyped a subset of individuals for the mitochondrial control region (CR; *n* = 66) and Z‐linked Aldolase B6 gene (*n* = 54). For the CR, we designed a primer set: Finch Siskin CR L85 (5′‐GGCACATCCTTGTTTCAGGT‐3′) and H807 (5′‐CAGTGCCAAGTTTGMGACGA‐3′) to amplify a 576 bp sequence and used the primer set previously published by Cheviron and Brumfield (2009) to amplify a 709 bp sequence for Aldolase B6.

Polymerase chain reactions (PCRs) were performed in a 25 µl reaction containing Green GoTaq® Flexi buffer (Promega), 0.2 mM dNTP, 2 mM MgCl_2_, 0.5 µM each primer, 0.5 U GoTaq® Flexi DNA polymerase (Promega). The profile for amplification of CR was an initial cycle of 2 min at 94℃, 45 s at 50℃, and 1 min at 72℃; then 31 cycles of 30 s at 94℃, 45 s at 50℃, and 60 s at 72℃; and one final extension at 72℃ for 5 min followed by 4℃ for 20 s. The same conditions were used for AldoB except 2.5 mM MgCl_2_ and an annealing temperature of 62℃. Amplified DNA was sequenced at Genome Quebec (Montreal, QC, Canada). Chromatograms were checked and visually aligned using MEGA 6.0 (Tamura et al., 2013).

### 2.3 Microsatellite genotyping

A subset of individuals was initially screened for amplification and variation with 26 passerine loci, 10 of which were designed for white‐crowned sparrows. In total, 12 loci produced PCR products, of which nine were polymorphic (Bensch et al., 1997; Dawson et al., 1997; Hanotte et al., 1994; Petren, 1998; Poesel et al., 2009; Stenzler et al., 2004). Genomic DNA was amplified in 10 µl reaction volumes containing colorless GoTaq® Flexi buffer (Promega), 0.2 mM dNTP, and 0.8 mM or 1 mM MgCl_2_, 0.5 µM forward and reverse primer, 0.05 µM fluorescent M13 tag, 0.5 U GoTaq® Flexi DNA polymerase (Promega). The amplification profile consisted of a 2‐min denaturation at 94℃, 45 s at 50℃, and 1 min at 72℃; seven cycles of: 1 min at 94℃, 30 s at T_m1_, and 45 s at 72℃; 31 cycles of; 30 s at 94℃, 30 s at T_m2_, and 45 s at 72℃; and a final 5‐min elongation at 72℃. Annealing temperatures (T_m1_/T_m2_) differed for each primer set: Gf06, Pocc2, & VeCr05 (45/47), ZoleH02 (50/52), Gf01 & ZoleA2 (55/57), and Escu6, YW16, & ZoleC11 (60/62). For Escu6, the second step was decreased from 31 to 25 cycles. Products were visualized on a 6% acrylamide gel using a LI‐COR 4300 DNA Analyser (LI‐COR Inc., Lincoln, NE, USA). Three positive controls of known sizes were included for each locus to ensure consistent scoring. Genotyping data were scored by two separate observers to ensure consistent scoring, and any discrepancies were checked. A subset of samples (~24) was genotyped a second time to ensure consistent scores across gels.

### 2.4 Genetic diversity analyses

Out of 54 birds used for AldoB6, 31 were male. Haplotypes from these males were reconstructed using PHASE in DnaSP v5 (Rozas et al., 2017) to account for the sex‐linked nature of AldoB6 resulting in 85 sequences. DnaSP v5 was used to calculate haplotype diversity (*H*_d_) for both AldoB6 and CR.

We constructed separate maximum‐likelihood haplotype networks for CR and AldoB6 to examine population structure among populations and subspecies. Networks were constructed using PopART 1.7 (Bandelt et al., 1999); each network was run for 500 iterations. Pairwise *θ*_ST_ values were calculated in Arlequin v3.5 and significance determined using 999 permutations (Excoffier & Lischer, 2010). All *p*‐values were corrected for multiple tests using the false discovery rate (FDR) (Benjamini & Yekutieli, 2005).

For microsatellite diversity analyses, all samples were checked for errors and null alleles with MICRO‐CHECKER v2.2.3 (Van Oosterhout et al., 2004). We tested for deviations from Hardy–Weinberg equilibrium and linkage disequilibrium using the default parameters in GENEPOP v4.2 (Rousset, 2008). We calculated observed and expected heterozygosities, number of alleles per locus, and private alleles for all 15 populations and pairwise *F*′_ST_ matrix and corresponding *p*‐values (Meirmans & Hedrick, 2011) in GenAlEx v6.5 (Peakall & Smouse, 2012). Additionally, we calculated pairwise *F*′_ST_ values among the four ecosites to quantify genetic differentiation between habitat types.

### 2.5 Assessing population genetic structure

STRUCTURE v2.3 is a nonspatial Bayesian clustering method which uses a Markov chain Monte Carlo (McMC) simulation to determine the number of genetic clusters (*K*) based on multilocus genotype data (Pritchard et al., 2000). The program was run with correlated allele frequencies and the admixture model with sampling locations as *locpriors*. Ten independent runs were performed with 50,000 burn‐ins and 200,000 McMC repetitions for each *K* value from *K* = 1–10. To determine the optimal number of clusters, STRUCTURE HARVESTER v0.694 (Earl & vonHoldt, 2012) was used to average values of LnPr(*X*|*K*) and Δ*K* for each *K* (Evanno et al., 2005). Seven of the ten northern populations showing admixture were run separately with the same settings to determine whether additional structure was present. To determine whether genetic structure corresponded to ecosite type (northern populations) or elevation (CO and OR), we used ecosite or elevation as *locpriors* and ran STRUCTURE using the same settings as above.

To complement our STRUCTURE analysis, we used a second Bayesian clustering algorithm TESS v2.3 (Chen et al., 2007) that incorporated spatial information. TESS was run for *K* values from 1 to 10 using 5,000 burn‐ins and 100,000 sweeps, with a spatial interaction parameter (Ψ) of 0.6. The optimal *K* was determined by choosing the *K* where the deviance information criterion (DIC) value began to plateau (Chen et al., 2007). As with STRUCTURE, the same groupings for investigating admixture, ecosite types, and elevation were run separately for *K* values from 1 to 6 with the same settings to uncover possible substructure.

### 2.6 Multivariate analyses

To further assess genetic structure from a multivariate perspective, Principal Coordinate Analysis (PCoA) and a Principal Component Analysis (PCA) were performed in R (R Core Team 2021). For the PCoA, a matrix of multivariate genetic distances (*F*′_ST_ values) between all populations was plotted against their geographic coordinates. A three‐dimensional PCoA graph was made using the Scatterplot3D package (Ligges & Mächler, 2003) to visualize the first three principal coordinates. In addition to comparing genetic differences, we also used multivariate analyses to examine morphological differences among ecotypes. We conducted a PCA on six morphological measurements: bill length, bill depth, bill width, tarsus length, uncompressed wing chord, and body mass. For this analysis, we compared morphological differences from three ecotypes: alpine coniferous, riparian deciduous, and disturbed‐gas site. Morphological differences were summarized with a standard multivariate ordination using the ggbiplot package (Wickham, 2009).

### 2.7 Species distribution models

We constructed a species distribution model (SDM) to display potential dispersal routes of white‐crowned sparrows using least‐cost path (LCP) and least‐cost corridor (LCC) models. The SDM was made in ArcMap 10.1 (ESRI, Redlands, CA) using SDMtoolbox v1.1 (Brown, 2014). We obtained 552 occurrences of *Z. l. gambelii*, 762 of *Z. l. oriantha*, and 1,285 of *Z. l. pugetensis* from the Global Biodiversity Information Facility (GBIF, 2018a, 2018b, 2018c; http://data.gbif.org/). Occurrences were limited to breeding season by filtering for data collected from mid‐April through August, and we restricted our occurrence data to points collected after 1980. We used environmental data from three separate sources: WorldClim Global Climate Dataset (v2, http://worldclim.org/version2), MODIS‐based Global Land Cover dataset (Broxton et al., 2014), and the Digital Elevation Model (DEM; resolution equals 1 km) developed by the USGS Earth Resources Observation and Science Center (EROS) (http://cec.org/tools‐and‐resources/map‐files/elevation‐2007). The WorldClim Global Climate Dataset summarizes variables that measure of precipitation and temperature from 1970 to 2000, while the MODIS‐based Global Land Cover dataset summarizes land cover categorizing land cover into 16 distinct classifications.

We used SDMtoolbox to spatially rarefy occurrence data to account for sampling bias; following this analysis, we were left with 216 occurrences for *Z. l. gambelii,* 285 for *Z. l. oriantha,* and 348 for *Z. l. pugetensis*. Next, the 19 environmental variables, land cover, and elevation layers were checked for autocorrelation and clipped to the extent of North America. Each subspecies' ecological niche was then modeled using Maxent v3.4 (Phillips et al., 2006) with a regularization multiplier of 2, 10 replicates of 500 iterations with cross‐validation using the elevation, land cover, and the resulting 10 uncorrelated environmental variables (annual mean temperature, mean diurnal range, isothermality, temperature seasonality, mean temperature of warmest quarter, annual precipitation, precipitation seasonality, precipitation of wettest quarter, precipitation of warmest quarter, precipitation of coldest quarter). With the exception of a regularization multiplier of 5 for *Z. l. gambelii*, the same variables and settings were used. Corrected Akaike's information criterion (AICc) and the area under curve (AUC) (Warren & Seifert, 2011) were used to select the best fit model.

### 2.8 Correlates of genetic structure with distance and dispersal

To model the most likely dispersal routes and dispersal costs, LCP and LCC analyses were conducted with SDMtoolbox v1.1c (Brown, 2014). We inverted our SDM to create a friction layer and used the Landscape Connectivity tool to create the sum of LCP and LCC between all population pairs. For the LCPs, we used the route with the lowest friction value between each population pair as our LCP measurement. Following the approach outlined by Jensen et al. (2019), we weighted the resistance values generated from our LCP analysis into three classes: low, mid, and high. These values are a “percentage of the LCP” where low, mid, and high represent 1%, 2%, and 5% of LCP values. Finally, we summed both the weighted and categorized LCP values to generate an LCC dispersal network. Because samples were from a small area of the breeding range, an LCC model was not made for *Z. l. pugetensis*.

### 2.9 Isolation by distance and resistance

We used Mantel and partial‐Mantel tests to examine the effects of isolation by distance (IBD) and isolation by resistance (IBR) on genetic differentiation. All Mantel and partial‐Mantel tests were conducted in GenoDive 3.04 (Meirmans & Van Tienderen, 2004). We converted pairwise *F*′_ST_ values to a distance measurement using the formula *F*′_ST_/(1 − *F*′_ST_) and calculated geographic distances between sites in GenAlEx 6.05 (Peakall & Smouse, 2012). For IBR, we generated a least‐cost path distance matrix using the along‐path cost of the LCP calculated in ArcMap 10.1. The along‐path cost is the total sum of the friction values that characterize the LCP and allows for the testing of IBR between sample sites (Etherington, 2011). We ran five IBD and IBR models: all populations, all populations excluding Oregon, only *Z. l. gambelii*, only *Z. l. oriantha*, and all northern Rocky Mountain populations. For each dataset, we also ran partial‐Mantel tests to examine the effect of IBD and IBR in the presence of each other to better determine which of these factors influences genetic patterns for white‐crowned sparrows.

## 3 RESULTS

### 3.1 Genetic diversity

The mitochondrial CR had a total of 17 haplotypes; six haplotypes were shared among multiple populations, while the remaining 11 haplotypes were restricted to a single population (Figure 2). Many of the northern individuals shared the same CR haplotype (71% of the 35 northern individuals), and most unique haplotypes from the northern populations originated from BA. Haplotype diversity was low for six populations (Hd = 0 in JAS, BV, LE, FTSJ, OK, and MK) and high for five (Hd > 0.7 in BA, WT, RV, CO‐H, and OR‐L; Table 1).

The Z‐linked AldoB6 locus had a total of 30 haplotypes, with eight shared among multiple populations and 22 restricted to a single population. The AldoB6 haplotype network showed some clustering of southern (OR and CO) and northern (AB and BC) populations. One common haplotype was found in 32% of 85 sequences from eight populations, mostly northern populations (Figure 2). AldoB6 haplotype diversity was low for three populations (Hd = 0 in LE, RV, and OK) and high for eight (Hd > 0.7 in BA, BV, WT, CO‐L, CO‐M, CO‐H, OR‐L, and OR‐H). The *Z. l. gambelii* subspecies had the lowest Hd at both loci (CR: 0.56, AldoB6: 0.84), *Z. l. pugetensis* had the highest Hd for CR (Hd = 0.83), and both *Z. l. pugetensis* and *Z. l. oriantha* are similarly high for AldoB6 (Hd = 0.90 and 0.91, respectively; Table 1).

All 328 individuals from 15 study sites were genotyped at nine microsatellite loci (Table 1). The average number of alleles per population ranged from 2.33 (OK) to 8.44 (OR‐L and OR‐H), observed heterozygosity (H_o_) ranged from 0.43 (OK) to 0.75 (JAS and CO‐H), and expected heterozygosity (He) from 0.39 (OK) to 0.73 (CO‐L and CO‐H; Table 1). Among the sample sites with the largest number of private alleles were WT and CO‐L with three, and OR‐H with four. The CO elevations had the highest level of allelic richness (4.32–4.56; Table 1).

Overall, we observed high population genetic structure and pairwise *θ*_ST_ values for CR data confirmed the separation of northern and southern populations (Table 2). Genetic differentiation between northern and southern populations was moderate to large (range: 0.10–0.50). By comparison, there was low to moderate genetic differentiation between all northern population pairwise comparisons, as well as southern population pairwise comparisons (range: 0–0.30). All three subspecies were genetically distinct based on control region sequences (range: 0.15–0.40). We observed low to high genetic variation for comparisons of AldoB8 sequences (range: 0–0.72). For the 15 AldoB8 *θ*_ST_ comparisons within northern populations, 46% were significant, and 20% were significant within the 10 southern population comparisons (Table 2). In the north, BV and MK accounted for all the significant differences and OR‐L in the south. Similarly, between the north and south, MK, LE, and OR‐L accounted for all but one of the significant results (Table 2). Among the three subspecies, *Z. l. oriantha* and *Z. l. gambelii* were significantly different from *Z. l. pugetensis*, but not from each other. Genetic divergence at this marker was relatively low (range: 0.04–0.12).

**TABLE 2:** Pairwise *θ*_ST_ values below diagonal and corresponding *p*‐values after FDR correction above diagonal for CR (top) and AldoB6 (middle)

CR														
	JAS	BA	BV	WT	LE	FTSJ	RV	OK	MK	CO‐L	CO‐M	CO‐H	OR‐L	OR‐H
^1^JAS	–	0.17	0.99	0.99	0.99	0.99	0.14	0.99	0.99	**0.03**	**0.03**	**0.03**	**<0.01**	**0.01**
^1^BA	0.22	–	0.63	0.30	0.37	0.17	0.32	0.63	0.63	0.06	**0.05**	**<0.01**	**<0.01**	**0.01**
^1^BV	0.00	0.00	–	0.99	0.99	0.99	0.26	0.99	0.99	**0.03**	**0.03**	**0.01**	**<0.01**	**0.03**
^1^WT	0.00	0.00	0.00	–	0.99	0.99	0.26	0.99	0.99	0.14	**0.13**	**0.04**	**<0.01**	**0.01**
^1^LE	0.00	0.15	0.00	0.00	–	0.99	0.26	0.99	0.99	**0.03**	**0.03**	**0.04**	**<0.01**	**0.02**
^1^FTSJ	0.00	0.22	0.00	0.00	0.00	–	0.14	0.99	0.99	**0.03**	**0.03**	**0.03**	**<0.01**	**<0.01**
^1^RV	0.30	0.04	0.09	0.19	0.21	0.30	–	0.33	0.33	**0.03**	**0.03**	**<0.01**	**<0.01**	**0.01**
^1^OK	0.00	0.02	0.00	0.00	0.00	0.00	0.06	–	0.99	0.07	0.07	0.11	**0.01**	**0.04**
^1^MK	0.00	0.02	0.00	0.00	0.00	0.00	0.06	0.00	–	0.07	0.07	0.11	**0.01**	**0.05**
^2^CO‐L	**0.89**	0.27	**0.09**	0.11	**0.87**	**0.89**	**0.30**	0.84	0.84	–	0.99	0.55	0.33	0.10
^2^CO‐M	**0.89**	**0.27**	**0.09**	**0.11**	**0.87**	**0.89**	**0.30**	0.84	0.84	0.00	–	0.40	0.15	0.10
^2^CO‐H	**0.48**	**0.37**	**0.19**	**0.16**	**0.43**	**0.48**	**0.44**	0.36	0.36	0.00	0.01	–	0.08	**0.04**
^3^OR‐L	**0.61**	**0.49**	**0.34**	**0.33**	**0.59**	**0.61**	**0.55**	**0.54**	**0.54**	0.28	0.08	0.09	–	0.80
^3^OR‐H	**0.78**	**0.32**	**0.17**	**0.19**	**0.74**	**0.78**	**0.36**	**0.69**	**0.55**	0.30	0.30	**0.19**	0.00	–

AldoB6											
	BA	BV	WT	LE	RV	MK	CO‐L	CO‐M	CO‐H	OR‐L	OR‐H
^1,2^BA	–	0.74	0.72	0.07	0.33	0.27	0.51	0.25	0.20	0.16	0.99
^2^BV	0.01	–	0.48	**<0.01**	**0.04**	**0.03**	0.50	**0.03**	0.11	0.06	0.60
^1,2^WT	0.00	0.01	–	0.43	0.39	0.04	0.99	0.32	0.43	0.14	0.43
^1^LE	0.72	**0.30**	0.17	–	0.07	**<0.01**	0.19	0.11	**<0.01**	**0.01**	**0.03**
^1^RV	0.50	**0.18**	0.25	1.00	–	**0.03**	0.17	0.08	0.44	**0.01**	0.06
^1,2^MK	0.02	**0.02**	0.08	**0.34**	**0.24**	–	**<0.01**	**<0.01**	**<0.01**	**<0.01**	0.20
^2^CO‐L	0.10	0.00	0.00	0.14	0.26	**0.08**	–	0.42	0.36	0.08	0.24
^2^CO‐M	0.19	**0.10**	0.07	0.37	0.45	**0.13**	0.00	–	0.22	**0.02**	0.10
^2^CO‐H	0.10	0.04	0.01	**0.39**	0.06	**0.08**	0.02	0.06	–	**<0.01**	0.12
^3^OR‐L	0.13	0.05	0.08	**0.31**	**0.33**	**0.10**	0.08	**0.16**	**0.14**	–	0.29
^3^OR‐H	0.00	0.00	0.08	**0.50**	0.31	0.02	0.08	0.15	0.08	0.04	–

CR			
	*Z. gambelli*	*Z. oriantha*	*Z. pugetensis*
*Z. gambelii*	–	**<0.01**	**<0.01**
*Z. oriantha*	**0.15**	–	**<0.01**
*Z. pugetensis*	**0.40**	**0.18**	–

AldoB6			
	*Z. gambelii*	*Z. oriantha*	*Z. pugetensis*
*Z. gambelii*	–	0.15	**<0.01**
*Z. oriantha*	0.04	–	**<0.01**
*Z. pugetensis*	**0.12**	**0.06**	–

Note

Superscripts indicate the subspecies found at each sampling site: ^1^ *Z. l. gambelii*, ^2^ *Z. l. oriantha*, and ^3^ *Z. l. pugetensis*.

We observed high population genetic structure based on microsatellite genetic patterns; pairwise *F*′_ST_ values from all 15 populations ranged from 0 (JAS:RV) to 0.58 (BA:CO‐M), and 94 out of 105 (89.5%) comparisons were significant after FDR at the *p* < .05 level (Table 3). All but three of the nonsignificant comparisons involved JAS or RV, which have smaller sample sizes (Table 1). Notably, JAS was not genetically different from RV, OK, or MK populations on the opposite side of the Rocky Mountains, but it was significantly different from four adjacent populations on the same side of the mountains (WT, LE, CNP, and BA).

**TABLE 3:** Pairwise *F*′_ST_ results from nine microsatellite loci for 15 populations below diagonal and corresponding *p*‐values after FDR correction above diagonal

	JAS	BA	BV	CNP	WT	LE	FTSJ	RV	OK	MK	CO‐L	CO‐M	CO‐H	OR‐L	OR‐H
^1^JAS	–	**<0.01**	0.11	**<0.01**	**<0.01**	**<0.01**	**<0.01**	0.43	0.31	0.31	**<0.01**	**<0.01**	**<0.01**	**<0.01**	**<0.01**
^1,2^BA	**0.35**	–	0.44	**<0.01**	0.09	**<0.01**	**<0.01**	**<0.01**	**<0.01**	**<0.01**	**0.02**	**<0.01**	**<0.01**	**<0.01**	**<0.01**
^2^BV	0.09	0.00	–	**0.01**	0.45	**<0.01**	**<0.01**	0.21	**0.03**	**<0.01**	**<0.01**	**<0.01**	**<0.01**	**<0.01**	**<0.01**
^2^CNP	**0.42**	**0.27**	**0.13**	–	**0.04**	**<0.01**	**<0.01**	**<0.01**	**<0.01**	**<0.01**	**<0.01**	**<0.01**	**<0.01**	**<0.01**	**<0.01**
^1,2^WT	**0.30**	0.07	0.00	**0.07**	–	**<0.01**	**<0.01**	**<0.01**	**<0.01**	**<0.01**	**<0.01**	**<0.01**	**<0.01**	**<0.01**	**<0.01**
^1^LE	**0.31**	**0.20**	**0.09**	**0.40**	**0.19**	–	**<0.01**	**<0.02**	**<0.01**	**<0.01**	**<0.01**	**<0.01**	**<0.01**	**<0.01**	**<0.01**
^1^FTSJ	**0.39**	**0.50**	**0.30**	**0.38**	**0.27**	**0.22**	–	**<0.01**	**<0.01**	**<0.01**	**<0.01**	**<0.01**	**<0.01**	**<0.01**	**<0.01**
^1^RV	0.00	**0.38**	0.07	**0.39**	**0.25**	**0.15**	**0.28**	–	0.45	0.44	**<0.01**	**<0.01**	**<0.01**	0.22	**<0.01**
^1^OK	0.04	**0.53**	**0.17**	**0.45**	**0.26**	**0.27**	**0.26**	0.00	–	**0.02**	**<0.01**	**<0.01**	**<0.01**	**<0.01**	**<0.01**
^1,2^MK	0.02	**0.24**	**0.11**	**0.30**	**0.26**	**0.23**	**0.34**	0.00	0.11	–	**<0.01**	**<0.01**	**<0.01**	**<0.01**	**<0.01**
^2^CO‐L	**0.33**	**0.10**	**0.13**	**0.37**	**0.20**	**0.18**	**0.41**	**0.35**	0.45	**0.33**	–	**<0.01**	**<0.01**	**<0.01**	**<0.01**
^2^CO‐M	**0.37**	**0.58**	**0.46**	**0.71**	**0.50**	**0.39**	**0.38**	**0.30**	0.25	**0.46**	**0.44**	–	**<0.01**	**<0.01**	**<0.01**
^2^CO‐H	**0.30**	**0.28**	**0.23**	**0.53**	**0.28**	**0.17**	**0.24**	**0.25**	0.27	**0.27**	**0.19**	**0.17**	–	**<0.01**	**<0.01**
^3^OR‐L	**0.17**	**0.16**	**0.11**	**0.36**	**0.18**	**0.11**	**0.33**	**0.05**	0.20	**0.17**	**0.22**	**0.37**	**0.23**	–	**0.04**
^3^OR‐H	**0.30**	**0.14**	**0.11**	**0.33**	**0.21**	**0.08**	**0.36**	**0.17**	0.36	**0.23**	**0.19**	**0.46**	**0.27**	**0.01**	–

Note

Superscripts indicate the subspecies found at each sampling site: ^1^ *Z. l. gambelii*, ^2^ *Z. l. oriantha*, and ^3^ *Z. l. pugetensis*.

### 3.2 Population genetic structure

Based on LnPr(*X*|*K*) and Δ*K*, *K* = 4 for microsatellite data from all 15 populations (Figure 3a). STRUCTURE identified four clusters: two in the north (a) JAS, RV, and MK; (b) BA, BV, CNP, WT, LE, FTSJ, and OK; one in the southeast Rocky Mountains, (c) CO‐L, CO‐M, and CO‐H; and another in the Pacific Northwest; and (d) OR‐L and OR‐H. The first two clusters were not as clearly defined as the other two. This admixture along with a bimodal Δ*K* plot prompted further hierarchical analyses to test for substructure within the northern samples. When the seven admixed northern populations (BA, BV, CNP, WT, LE, FTSJ, and OK) were analyzed separately, the result was *K* = 2; however, no clear geographic pattern emerged (Figure 3b). No substructure was found within either the CO or OR elevational transects. Ecosite analyses of all ten northern populations showed *K* = 2 with substructure corresponding to ecotypes: AC ecosite (JAS, BA, RV, MK, and some OK) and RD, D‐G, and D‐T ecosites (BV, CNP, WT, LE, FTSJ, and some OK; Figure 3c).

Using a spatial Bayesian clustering analysis in TESS (Figure 3d), the optimal number of clusters was *K* = 3 as determined by the highest DIC value. The clusters were similar to the four main clusters in STRUCTURE except OR grouped with some northern populations: (a) JAS, RV, OK, and MK; (b) BA, BV, CNP, WT, LE, FTSJ, OR‐L, and OR‐H; and (c) CO‐L, CO‐M, and CO‐H. When running each of the three clusters independently, the DIC values indicated *K* = 2 within each group: (a) JAS, RV, OK, and (b) MK for the first group, (c) BA, BV, CNP, WT, LE, and FTSJ, and (d) OR for the second group, and (e) CO‐L, CO‐H, and (f) CO‐M for the third group (Figure 3e). TESS uncovered substructure when testing for elevational differences in the CO sample sites that STRUCTURE did not. When northern populations were sorted by ecosite types, *K* = 2, again separating the alpine coniferous (AC) ecosite (JAS, RV, OK, and MK) from riparian deciduous (RD), disturbed‐gas (D‐G), and disturbed‐townsite (D‐T) ecosites (BA, BV, CNP, WT, LE, WT, OK, and FTSJ). Unlike STRUCTURE, in TESS, BA clusters with the RD, D‐G, and D‐T group of ecosites not the AC ecosite.

The PCoA based on *F*′_ST_ values showed the first two axes account for 34.5% and 23.6% of the variation with the third axis explaining 16.1% (Figure S1). When all three axes were displayed in a three‐dimensional plot, similar groupings observed in the other analyses were produced. The southern populations, CO and OR, separated from the northern populations. JAS was distinct from all the other AB populations, and OK, MK, and FTSJ clustered together and the remaining northern populations formed a single cluster.

**FIGURE 4:**
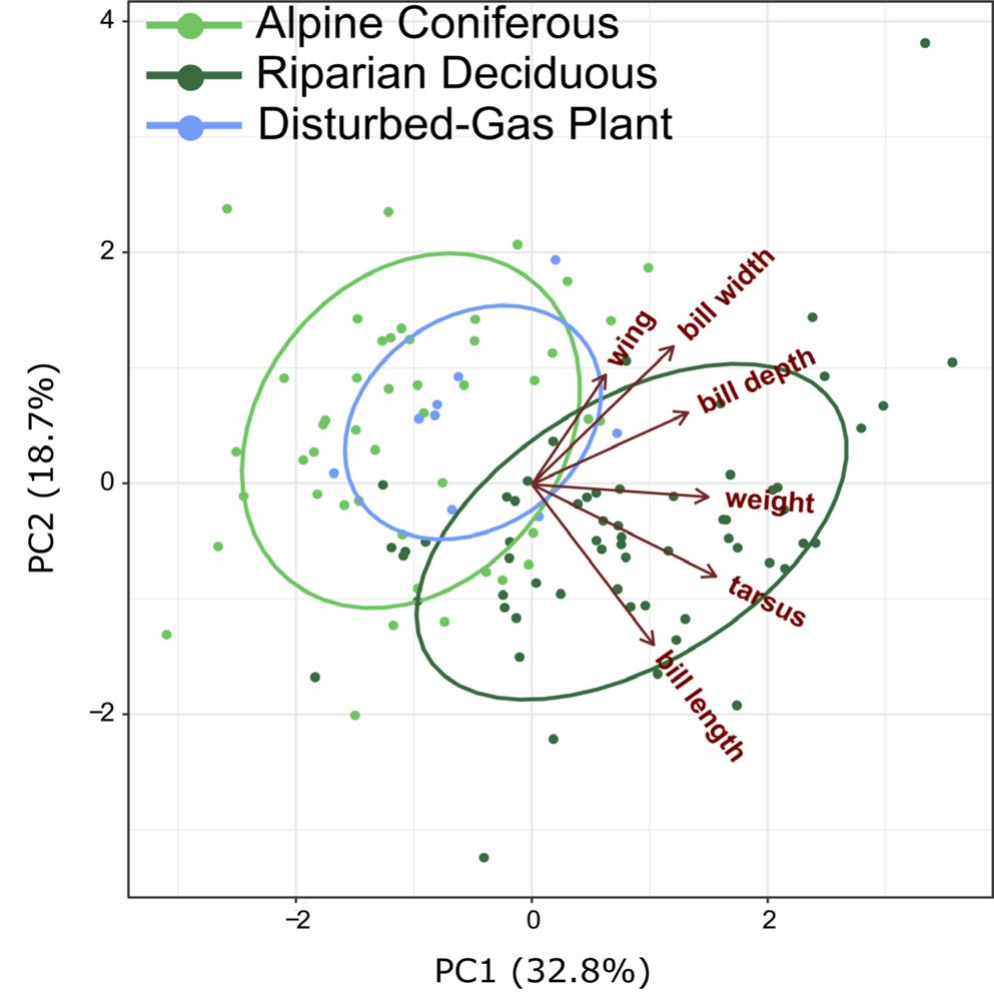
A standard principal component analysis of 113 individuals with multivariate analysis of six morphological features (wing length; bill width, length, and depth; tarsus length; and body weight). Ellipses show individuals from alpine coniferous (light green), riparian deciduous (dark green), and disturbed habitat (blue) ecosites as compared in TESS and STRUCTURE (Figure 3). Disturbed habitat individuals are from the disturbed‐gas plant site only, as no morphological data were available for the 11 townsite individuals, and CO and OR populations were omitted

The PCA using morphometric data from all northern populations showed two distinct ecosite groupings similar to STRUCTURE and TESS with the first and second axes accounting for 32.8% and 18.7% of the variation, respectively. When grouping individuals by ecosites, the six phenotypic traits clustered into AC and RD/D‐G ecosites. The samples from the disturbed group only include the D‐G population in CNP (Figure 4).

### 3.3 Species distribution models and potential dispersal routes

**FIGURE 5:**
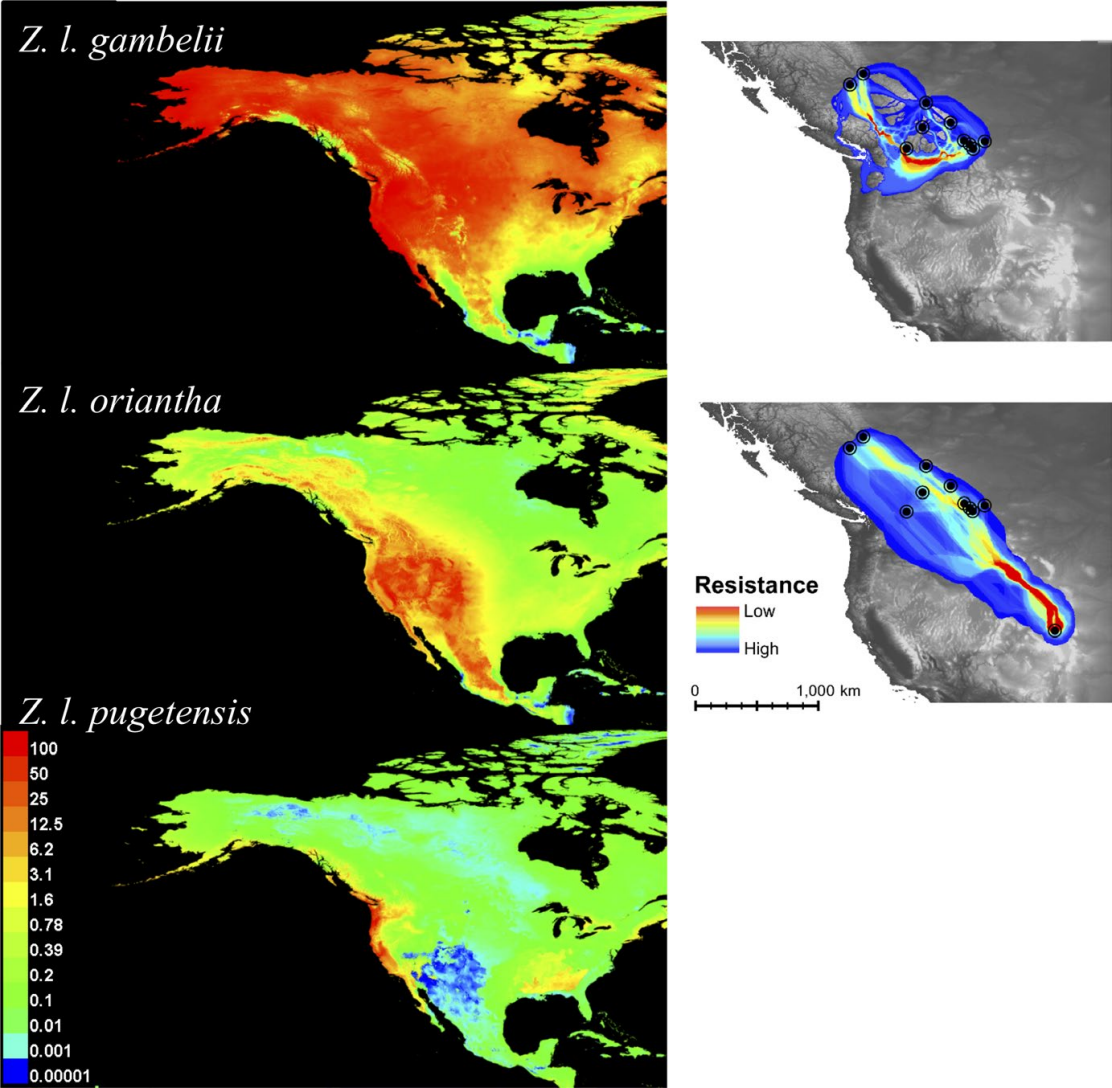
Contemporary SDM created using occurrence data from three subspecies of white‐crowned sparrow, environmental variables, a vegetation cover layer, and elevation layer. Areas of the most suitable environmental and habitat conditions (i.e., ecological niche) for each subspecies are in warmer colors (left). Least‐cost corridor (LCC) projections of dispersal routes between sites within the breeding ranges of *Z. l. gambelii* and *Z. l. oriantha* based on the SDMs with base maps showing higher elevations in lighter gray. Dispersal costs are coded by red representing areas with low resistance (i.e., low dispersal cost), and blue representing high resistance (right). The models were created using the SDM toolbox (Brown, 2014) in Arc GIS® and MaxEnt (Phillips et al., 2006)

The contemporary SDM for each subspecies closely followed their known distributions in North America (Figures 1 and 5). The variables with the greatest contributions were annual mean temperature (29.4%), followed by isothermality (15.7%), elevation (14.3%), temperature seasonality (12.6%), and precipitation of warmest quarter (12.2%) for *Z. l. gambelii*; elevation (71.7%), precipitation of warmest quarter (10.6%), isothermality (8.2%) for *Z. l. oriantha*; and precipitation of coldest quarter (61.9%), precipitation seasonality (10.5%), precipitation of warmest quarter (10.5%), and elevation (8.2%) for *Z. l. pugetensis* (Supporting Information). The layers contributing to the SDM for *Z. l. gambelii* yielded an AUC value of 0.828, where 0.5 means the model fit is no better than random, and values close to 1 are a good fit. The layers contributing to the SDMs for *Z. l. oriantha* and *Z. l. pugetensis* yielded high AUC values of 0.996 and 0.995, respectively.

The LCP and LCC for *Z. l. gambelii* showed dispersal routes with the lowest resistance were between mountain ranges in BC and through the Rockies in southwestern AB and southeastern BC. The southern Alberta *Z. l. gambelii* populations of LE, WT, CNP, and BV were connected via low elevation dispersal routes through the mountains to OK. Similarly, corridors connecting JAS to sites in BC had lower resistance than the corridors to other AB sites. Fort St. James and MK also had low resistance pathways connecting to OK, but not directly to each other. Temperature was the strongest contributor to the *Z. l. gambelii* SDM, yet dispersal patterns appeared to correspond to elevation. The *Z. l. oriantha* model showed low resistance north–south routes between CO, through the southern Alberta sites and extended up to MK along the lower elevation eastern foothills of the Rocky Mountains, a pattern that is corroborated by the importance of elevation as a contributor to this subspecies' SDM.

### 3.4 Correlates of genetic differentiation with distance and dispersal

For our models examining all populations excluding the two Oregon populations, IBD and IBR were significant (range: *r* = .24–.29, *p* ≤ .05). We were unable to separate the effects of these two processes, IBD and IBR, from each other (all partial‐Mantel tests *p* > .30). Among *Z. l. oriantha* and *Z. l. gambelii* populations, IBD and IBR were not significant. Additionally, IBD and IBR did not significantly affect genetic patterns among all Rocky Mountain populations.

## 4 DISCUSSION

We compared the influences of barriers and ecotype on population genetic structure at various spatial scales, and we hypothesized that geographic distance and mountains would act as barriers to gene flow within and among three subspecies of white‐crowned sparrows. Although IBD and IBR influence rangewide patterns, genetic patterns at local scales were not influenced by these factors. Our results indicate a role for local ecological factors and microclimate acting as barriers and influencing both rangewide and local genetic patterns.

### 4.1 Macrogeographic barriers: distance and hybrid zones

Physical distance played a large role in describing the rangewide genetic differentiation in *Z. l. gambelii* and *Z. l. oriantha* (Figure 2, Table 4). Within each of these ranges, large amounts of suitable habitat are present; however, habitat and dispersal are not homogenous as evident by the genetic analyses. Both nuclear datasets showed north–south differences and evidence of dispersal across mountain ranges (Table 3, Figure 2). The genetic similarity of JAS to RV, OK, and MK corresponds to the Yellowhead Pass, a major low elevation corridor through the Rocky Mountains used for dispersal by other species including mountain pine beetles (*Dendroctonus ponderosae*, Robertson et al., 2009), ruffed grouse (*Bonasa umbellus*, Jensen et al., 2019), and mountain caribou (*Rangifer tarandus caribou*, Hooge, 2003). Similarly, the genetic similarity of BV and RV could potentially be explained by dispersal through the Rocky Mountain Trench.

**TABLE 4:** Mantel tests comparing genetic distance with both geographic distance and dispersal resistance

Comparisons	IBD	IBR	IBD|IBR	IBR|IBD
**Across breeding range**	**r = .28**	***r* = .26**	*r* = .12	*r* = .01
(All fifteen sites included)	***p* = <.03**	***p = *.04**	*p* = .23	*p* = .45
**Sites within *Z. l. oriantha* breeding range**	***r* = .29**	***r* = .24**	*r* = .20	*r* = −.09
(Thirteen sites excluding OR)	***p = *.03**	***p* =.05**	*p* = .11	*p* = .30
***Z. l. gambelii***	*r* = −.01	*r* = .03	*r* = −.13	*r = .13*
(BA, WT, LE, FTSJ, RV, OK, MK)	*p = *.52	*p = *.52	*p* = .34	*p* = .30
***Z. l. oriantha***	*r* = .30	*r* = .25	*r* = .27	*r* = −.22
(BA, BV, CNP, WT, MK, CO‐L, CO‐M, CO‐H)	*p* = .14	*p* = .17	*p = *.18	*p* = .28
East and West of Rockies	*r* = .15	*r* = .17	*r* = −.08	*r = .11*
(JAS, BA, BV, CNP, WT, LE, FTSJ, RV, OK, MK)	*p* = .20	*p = *.18	*p = *.35	*p* = .28

Note

*F*′_ST_ values are compared against Euclidian distances between populations for a test of isolation by distance (IBD), and against the least‐cost path resistance values for a test of isolation by resistance (IBR). Significant *p*‐values are in bold. Variables following vertical lines indicate the variable that was controlled for during partial‐Mantel tests.

The differentiation of our CO samples from the northern individuals using microsatellite data may be due to the sampling gap or hybrid zones. Within the Rocky Mountain area of AB and BC, a hybrid zone exists between *Z. l. oriantha* and *Z. l. gambelii*, whereas in CO they are pure *Z. l. oriantha* (Figure 1). In the sampling gap between northern and CO sparrows, there could be patterns of introgression defining the hybrid zone edge. Alternatively, if there is clinal variation in ecosystems, the differentiation of northern populations of CO could be an artifact of that sampling gap (Barton & Hewitt, 1989). Exploring these two possibilities is important because the patterns of population differentiation within the hybrid zone do not correspond to subspecies, but to ecosystems.

### 4.2 Microgeographic barriers: Forest type and disturbed ecosystems

We found genetic clusters corresponding to alpine coniferous (AC) and riparian deciduous (RD) ecosite types and individuals from disturbed ecosites grouped with the riparian deciduous individuals (Figure 3). Morphological and genetic differences have been observed across heterogeneous landscapes in plants (Gram & Sork, 2001), insects (Hamer et al., 2003), aquatic invertebrates (Etter et al., 2005), and terrestrial vertebrates (Barley et al., 2015). Local environmental conditions and ecosystem have an important role in determining population genetic structure of white‐crowned sparrows. Individual white‐crowned sparrows from the same sampling area in OK belong to different genetic groups corresponding to the ecosite they were sampled in, although due to the low number of individuals collected from this area, our results should be viewed conservatively. No reports exist on the contemporary status of white‐crowned sparrows in the OK area. Brooks (1909) and Kermode (1904) report *Z. l. gambelii* breeding in this area, but other studies (Krannitz, 2007; Krannitz & Rohner, 2000) claim white‐crowned sparrows are transient. Our museum samples lacked specific data on indicators of breeding (i.e., brood patch or enlarged cloaca) and detailed collection dates. It is possible that our OK samples were migrating birds, but more sampling is required to ascertain whether white‐crowned sparrows breed in OK and have this interesting partition of genetic structure by ecosystem, especially considering the possibility of notable range shifts as have been observed in *Z. l. pugetensis* in the last 35 years (Hunn & Beaudette, 2014).

Ecosite type also corresponded to morphological differences in white‐crowned sparrow, albeit not by subspecies phenotype specifically (Figure 3). Birds found in riparian deciduous forests were larger than alpine coniferous birds (Figure 4). The same phenomenon of larger body size in deciduous compared with coniferous ecosystems was observed in male pied flycatchers (*Ficedula hypoleuca*) resulting from competition (Lundberg et al., 1981). The clustering of white‐crowned sparrows both genetically and morphologically into coniferous or deciduous forests is surprising. Most descriptions of white‐crowned sparrow habitat include thickets and streamside shrubs (*Z. l. gambelii*), meadows of high sagebrush near the conifer timberline (*Z. l. oriantha*), and early seral coniferous forest (*Z. l. pugetensis*) (Dunn et al., 1995; Morton, 2002; Rivers et al., 2019) rather than along deciduous forest edge as we observed for some of our samples. Since aspen understory and canopy support greater invertebrate abundance than conifers (Rumble et al., 2001), it is possible that larger body size of white‐crowned sparrows in deciduous forests is due to a greater abundance of high‐quality food. Similar genetic patterns were found in black‐capped chickadees, *Poecile atricapillus*, from the same area where patterns of genetic differentiation correspond to riparian poplar species and their hybrids (Adams & Burg, 2015). Body size may have also covaried with elevation, since 30% of our conifer forest samples were also from high elevations (>1,000 m). The greater energy costs of living in harsher climates of the high elevation sites could explain the smaller body size of sparrows sampled in those areas (Bonier et al., 2014). Although morphological patterns are congruent with genetic patterns, we cannot rule out the role of plasticity influencing morphological patterns. Therefore, we suggest that future studies should incorporate next‐generation sequencing techniques to further explore the relationship between morphological variation and genetic variation.

Forestry operations may be another influence on genetic structure in white‐crowned sparrows. Large forestry operations in the FTSJ area have been conducted since the 1950s (Proulx & Kariz, 2005). Microsatellite analyses grouped FTSJ with populations east of the Rockies despite it being (~400–600 km) northwest. White‐crowned sparrows establish territories quickly in recently harvested clear‐cuts (Hunn & Beaudette, 2014; Rivers et al., 2019). A population could have colonized the FTSJ clear‐cuts and subsequently experienced a founder effect from settling in habitats isolated from other populations, thus leaving a pocket of eastern alleles on the west of the Rockies. FTSJ did show reductions in genetic diversity relative to populations with similar sample sizes at the microsatellite loci (Table 1). Chickadees (*Poecile* spp.) from the same area show unusual genetic patterns. Hybrids of mountain (*Poecile gambeli*) and black‐capped chickadees are more abundant in the FTSJ area (Grava et al., 2012), and populations of black‐capped chickadees show high levels of population genetic structure (Adams & Burg, 2015).

### 4.3 Microgeographic barriers: Elevation

Differences in vegetation, elevation, and climate are highly restrictive in many species (Coulon et al., 2004; DuBay & Witt, 2014; Dubey et al., 2011; Funk et al., 2005; Gonzalo‐Turpin & Hazard, 2009; Lee‐Yaw et al., 2009; Olah et al., 2017), and white‐crowned sparrows are no exception. Several, more sensitive analyses using *F*′_ST_ comparisons from microsatellite data (Table 3, Figure 3) were able to detect significant differentiation between the proximate populations along elevational transects in CO. To access CO‐M from CO‐L requires passage from a river valley of rich biodiversity and plentiful shrub nesting habitat, through a long, narrow, and barren mountain pass. The CO‐M site is a unique and isolated meadow of short willow and open grassy areas surrounded by steep and densely forested mountain slopes. The CO‐H site is in neighboring alpine meadows far upslope. This local differentiation could be due to habitat differences, elevation, or both. Chamberlain et al. (2016) modeled the distributions of ten alpine bird species along elevation gradients and found that adding ecosystem data (vegetation cover and topography details) along with the climate and elevation variables improved model performance significantly for many species, reinforcing our observations that ecosystem is a key contributor to white‐crowned sparrow population structure. The OR sites only appear to have genetic differentiation in pairwise *F*′_ST_ analysis (Table 3), which may be due to the small elevational difference (430 m), or because all samples were from disturbed ecosystem (clear‐cuts). To detect whether elevation causes microgeographic structure as we hypothesized may require larger elevational differences.

### 4.4 Species distribution models and landscape resistance

SDMs and LCCs showed that whereas elevation is an important contributor to modeling suitable habitat for three white‐crowned sparrow subspecies, the Rocky Mountain range is a porous landscape barrier, especially for *Z. l. gambelii*. There is a lack of conspicuous barriers and a high degree of habitat suitability encompassing our *Z. l. gambelii* sample sites and much of the rest of North America (Figure 5) which could explain why geographic distance (IBD) instead of landscape resistance (IBR) is the most prevalent barrier to dispersal (Table 4). IBD also explains most of the genetic distance between *Z. l. oriantha* populations, but unlike in *Z. l. gambelii,* IBR had small but significant importance at various spatial scales in *Z. l. oriantha* (Table 4). This difference may exist because *Z. l. gambelii* is a habitat generalist while *Z. l. oriantha* is more of a habitat specialist (Hahn et al., 1995; Maney et al., 1999; Wingfield et al., 1996). *Z. l. oriantha* show robust responses to temperature and snowpack conditions in montane ecosystems, have strong site‐fidelity, and require specific conditions for nesting (Wingfield et al., 2003). *Z. l. gambelii* are “spatial opportunists” and show low site‐fidelity (Hahn et al., 1995). This phenomenon is reflected in sympatric species of butterflies, in which the genetic structure of the generalist species was unaffected by the landscape matrix; however, the specialist species were highly sensitive to fine‐scale ecosystem features (Engler et al., 2014).

In contrast to *Z. l. oriantha* and *Z. l. pugetensis*, the wider distribution of *Z. l. gambelii* could also be a result of being historically established on the east side of the Rocky Mountains where there are fewer physical barriers. This pattern is mirrored in many other species of high‐latitude boreal birds where divergence of sister‐species has been linked to fragmentation of the boreal forest by ice sheets in the Middle and Late Pleistocene (Weir & Schluter, 2004). The Taiga, Rocky Mountain, and Pacific Coast distributions of eight sets of sister‐species from Weir & Schluter's study (2004) follow a very similar distribution to the white‐crowned sparrow subspecies in our study. Alternatively, the current subspecies distributions could be the result of recent divergence due to local adaptation (Richardson et al., 2014). Our results highlight the importance of ecosystem in contemporary genetic differentiation (Figures 3 and 4), and LCC models show each subspecies' distribution is affected differently by environmental variables (Figure 5). Temperature is the strongest contributor for the *Z. l. gambelii* SDM, precipitation for *Z. l. pugetensis*, and elevation is important in the distribution models of all three subspecies, especially *Z. l. oriantha*. Further research is required to elucidate whether the current subspecies distributions are the result of historical range expansion (Banks, 1964; Rand, 1948), habitat fragmentation (Weir & Schluter, 2004), or recent divergence due to local adaptation of this ubiquitous species (Richardson et al., 2014).

### 4.5 Genetic variation among subspecies

Genetic partitioning among subspecies of white‐crowned sparrows has been previously established (MacDougall‐Shackleton & MacDougall‐Shackleton, 2001), and results indicate genetic partitioning among the subspecies investigated in this study provide further support for these patterns. Control region sequences indicate that *Z. l. gambelii* are distinct from *Z. l. oriantha* populations, although genetic variation between subspecies is relatively subtle. Only two control region haplotypes are shared between these two subspecies, and these haplotypes are found in southern Canada in the putative contact zone between these two subspecies. *Z. l. pugetensis* is also distinct from *Z. l. gambelii*, but shares several haplotypes with *Z. l. oriantha*. Microsatellite genetic patterns also indicate genetic differentiation between the three subspecies, and again individuals with admixed genotypes are found in southern Alberta where subspecies come into contact. Although other studies have found limited support for genetic distinctiveness among subspecies (Ball & Avise, 1992; Zink et al., 2000), genetic patterns do not always follow subspecies boundaries (Zink, 2004). The prevalence of genetic partitioning among the subspecies investigated in this study is further evidence of how complex genetic patterns are for this species and indicates that current taxonomy should continue to recognize the existing subspecies.

## 5 CONCLUSION

In conclusion, it is important to understand all the influences and barriers on population genetics in montane systems as they are an important ecological zone for biodiversity (Fjeldsâ et al., 2011). The western extent of the white‐crowned sparrow's distribution is genetically structured not by subspecies, but by the combination of distance, mountains, and ecotype. White‐crowned sparrows showed both genetic differentiation and morphological differentiation in coniferous versus deciduous ecosystems, a phenomenon of habitat partitioning increasingly observed in other population genetics studies (Grava et al., 2012; Jensen et al., 2019; Lundberg et al., 1981; Porlier et al., 2012). Using a widespread and ubiquitous avian species can contribute to an understanding of barriers that could be affecting the gene flow in species of conservation concern which are more difficult to access. For populations of threatened species isolated after habitat fragmentation and degradation, using a model species to understand the factors affecting dispersal and gene flow can be critical for conservation and management decisions (Dubey et al., 2011).

## CONFLICT OF INTEREST

None declared.

## AUTHOR CONTRIBUTIONS

**Catherine A. Welke:** Conceptualization (lead); formal analysis (lead); investigation (lead); methodology (lead); writing‐original draft (lead). **Brendan Graham:** Data curation (equal); writing‐review & editing (equal). **Ross R. Conover:** Funding acquisition (equal); methodology (equal); writing‐original draft (equal); writing‐review & editing (equal). **James W. Rivers:** Data curation (equal); funding acquisition (equal); methodology (equal); writing‐original draft (equal); writing‐review & editing (equal). **Theresa M. Burg:** Conceptualization (equal); data curation (equal); funding acquisition (equal); investigation (equal); methodology (equal); project administration (equal); supervision (equal); writing‐original draft (equal); writing‐review & editing (equal).

## Supporting information

Fig S1

Fig S1‐legend

## Data Availability

All data are archived in Dryad https://doi.org/10.5061/dryad.zw3r22882.
