# Supplementary figures and images for: Habitat‐linked genetic structure for white‐crowned sparrow (Zonotrichia leucophrys): Local factors shape population genetic structure
Source: Ecol Evol. 2021 Aug 10;11(17):11700–17. doi: 10.1002/ece3.7887 (PMC8427623; doi:10.1002/ece3.7887)

PCo3 (16.13%)

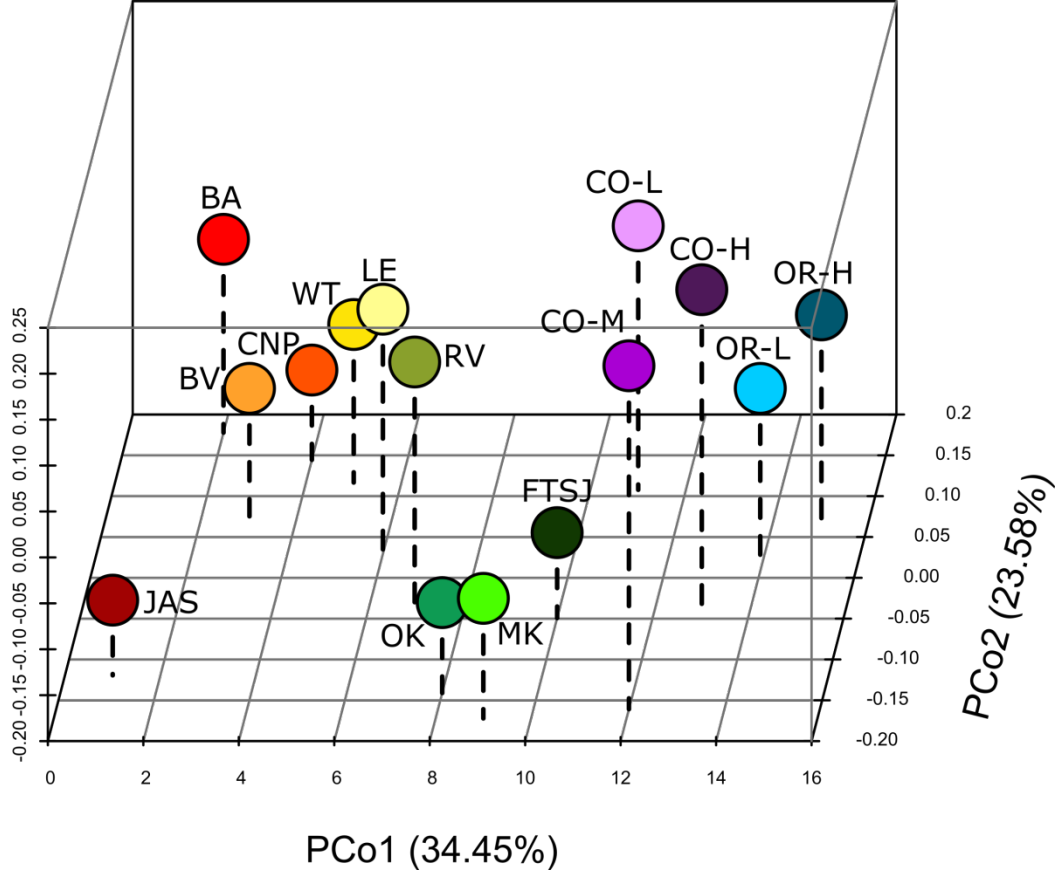

PCo1 (34.45%)

PCo2 (23.58%)

Supplement: Supplementary file 1 — Fig S1 [file ECE3-11-11700-s002.pdf]
