# Supplementary material for: Habitat‐linked genetic structure for white‐crowned sparrow (Zonotrichia leucophrys): Local factors shape population genetic structure
Source: Ecol Evol. 2021 Aug 10;11(17):11700–17. doi: 10.1002/ece3.7887 (PMC8427623; doi:10.1002/ece3.7887)
Supplement: Supplementary file 2 — Fig S1‐legend [file ECE3-11-11700-s001.docx]

**Figure S1:** A three-dimensional plot of the first three axes of the principal coordinate analysis of all 15 populations using multilocus genotype data.
